# High-resolution dynamic inversion imaging with motion-aberrations-free using optical flow learning networks

**DOI:** 10.1038/s41598-019-47564-z

**Published:** 2019-08-05

**Authors:** Jin Li, Zilong Liu

**Affiliations:** 1 Division of Optics, National Institute of Metrology, Beijing, 100029 China; 2 Electrical Engineering Division, Department of Engineering, University of Cambridge, Cambridge, UK

**Keywords:** Imaging and sensing, Aerospace engineering

## Abstract

Dynamic optical imaging (e.g. time delay integration imaging) is troubled by the motion blur fundamentally arising from mismatching between photo-induced charge transfer and optical image movements. Motion aberrations from the forward dynamic imaging link impede the acquiring of high-quality images. Here, we propose a high-resolution dynamic inversion imaging method based on optical flow neural learning networks. Optical flow is reconstructed via a multilayer neural learning network. The optical flow is able to construct the motion spread function that enables computational reconstruction of captured images with a single digital filter. This works construct the complete dynamic imaging link, involving the backward and forward imaging link, and demonstrates the capability of the back-ward imaging by reducing motion aberrations.

## Introduction

**Figure 1:**
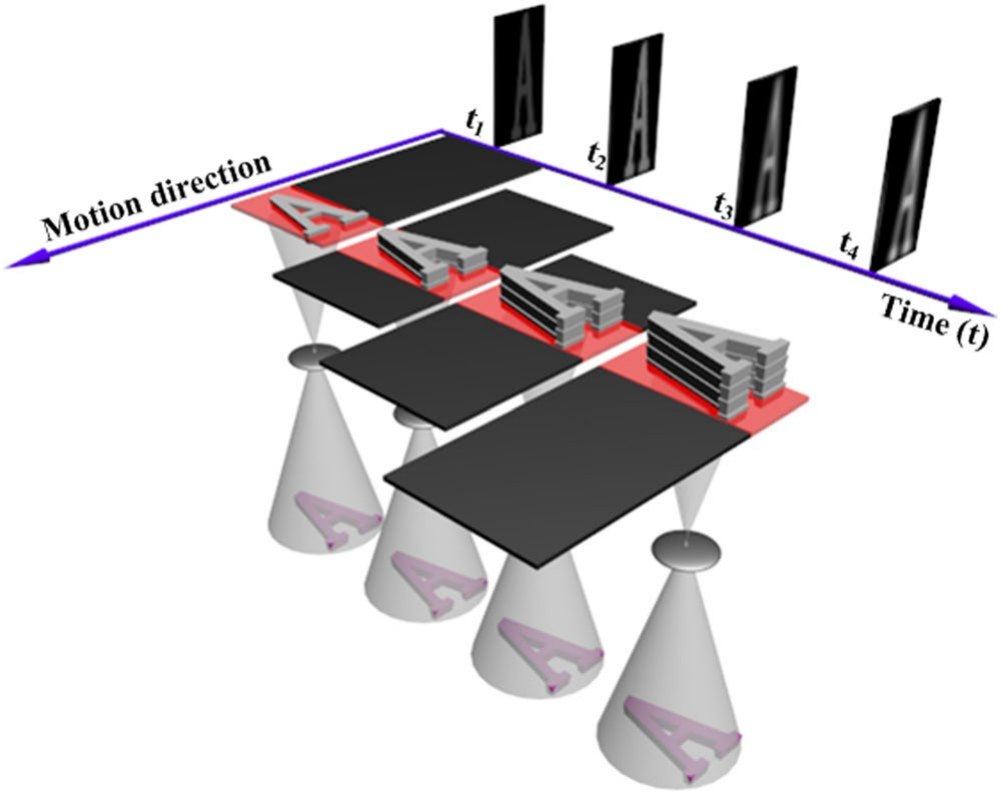
Dynamic imaging principle including the four integration times. A photoreception and a optical system sequentially moves with a stationary object. Meanwhile, photo-induced charges are transferred from one stage to the next stage.

Dynamic optical imaging is able to acquire images in the motion condition either a moving camera observes a stationary scene, or a stationary camera observes a moving scene, or a moving camera observes a moving scene. Dynamic optical imaging has been widely used in many fields, especially in low-light-level imaging based on time delay integration (TDI) technology of Charge Coupled Device (CCD) or Complementary Metal–Oxide–Semiconductor (CMOS)^1–6^. Dynamic optical imaging fundamentally involves two movements: photo-induced charge transfer and optical image movements. Figure 1 shows a typical dynamic imaging principle with time-delay integration.

Optical images are formed when lights of objects pass through the optical system of a camera. In the dynamic condition, Optical images gradually move on the photoreception. In the meanwhile, the photo-induced charge is gradually transferred and accumulated from the previous stage to the current stage. The current photo-induced charge is the sum of the previous stages with the current stage. After the integration period of the last stage, the photo-induced charge packet accumulated by the multiple integration stages is transferred a horizontal register and then is output and processed by an analog-digital converter to form a line image. The relative movement between photo-induced charges and optical images inevitably appear motion aberrations in the dynamic imaging process.

To remove motion aberrations (i.e. compensation for the motion blur) in the dynamic imaging process, two forward imaging approaches (i.e. forward active imaging approaches and forward passive imaging approaches) have been developed. The forward active imaging approaches aim at the matching between the photo-induced charge transfer speed and direction and the optical image motion velocity and direction to obtain high-quality images. Any mismatching of movement vectors (i.e. speed and direction) between the photo-induced charge transfer and optical image movement in dynamic processing would produce motion aberrations and the corresponding image is blurred. Forward active imaging approaches firstly calculate the motion vector of optical images based on the geometric imaging relationships^7–12^ between cameras and objects. The motion vector calculation needs to use auxiliary information from multi-sensory measurements, involving position and attitude sensors^13^ in conjunction with the imaging equations of the camera^14^. The forward active imaging approach enables matching between the image motion vector and photogenic charges transfer to compensate for motion blur. The speed component of motion vectors is used as the reference to control the integration time^15^ and the direction component adjusts the image sensor direction by means of mechanical structures (i.e. angle drift mechanism)^16^. However, the motion vector is not completely accurate because of motion vector model errors and the measurement errors of sensors^17^. Moreover, the measurement frequency of current attitude sensors is far lower than the photogenic charges line-transfer frequency. Optical image movements are achieved by angle drift mechanisms in conjunction with electronic controlling, where a lot of errors (e.g. controlling errors, mechanical errors, assembly errors, measurement errors, etc.) exist. Therefore, residual motion aberrations of the forward active imaging method disturb dynamic imaging and residual motion blur still exist in acquired images.

Forward passive imaging methods firstly introduce a high-speed motion sensor in the optical camera to measure images^18–20^. After that, optical correlators are utilized to calculate the relative motion between the photo-induced charge and the optical image, enabling compensation for image motion blur. Optical correlators are able to be achieved by block-matching methods^21–23^ to measure image motion to avoid the active imaging problem arising from multisensory measurement errors, controlling errors, complex imaging model errors, etc. However, forward passive imaging methods need to transform the motion information of the high-speed motion sensor to the camera. The transform errors inevitably exist because of position measurement errors and assembly errors of motion sensors. Therefore, forward passive imaging methods inevitably involve the mismatching (either motion speed, direction, or both) between the photo-induced charge transfer and optical image movement, which means motion aberrations appear in the dynamic imaging process.

To remove the motion aberrations from the forward imaging, backward inversion imaging methods are very interesting. Wang *et al*. proposed an active optical flow method to remove the residual high-frequency motion aberration of the forward active imaging approaches^24^. However, the optical flow has low inversion accuracy because auxiliary parameters used in optical flow inversion, such as the attitude and position information from the multiple sensory, has a lot of measurement errors. Computational imaging methods including blind deconvolution (BD) approach^25–29^ and modulate transfer function compensation (MTFC) method^30–32^ are able to be used in the backward inversion imaging. Unfortunately, the BD-based methods suffer from high computational complexity because it doesn’t utilize any prior knowledge of dynamic imaging. The MTFC-based methods are suitable to compensate for the total degraded factors including a camera optical system, image sensor, atmospheric, electronic circuits, atmosphere, temperature, etc.

Here, we demonstrate an efficient dynamic backward inversion imaging method with optical flow deep learning to remove motion aberrations. The proposed method uses deep learning networks^33–35^ to construct optical flow which enables reconstruction of the motion point spread function that is used to recover the observed image in dynamic forward imaging link.

## Proposed Method

### Forward imaging model

The image motion velocity vector (denoted by Ф) of the optical image formed by lights passing optical systems can be characterized by amplitude-phase form as

1 $${\rm{\Phi }}=K({v}_{P},\beta ),$$

2 $${v}_{P}=\sqrt{{({v}_{{P}_{x}})}^{2}+{({v}_{{P}_{y}})}^{2}},$$

3 $$\beta =\arctan (\frac{{v}_{{P}_{y}}}{{v}_{{P}_{x}}}),$$

where *v*_*p*_ is the amplitude of the image motion velocity vector, *β* is the phase (i.e. drift angle) of the image motion velocity vector, (*v*_*px*_, *v*_*py*_) are vertical and horizontal components, *K* is the coordinate transform matrix between the motion sensor and image sensor of the camera in the forward passive imaging. In the forward active imaging, *K* = [1 0; 0 1]. The drift angle *β* represents the direction of motion velocity vector of optical images on the focal plane, while the velocity *v*_*p*_ represents the motion velocity in the current direction *β*. To match between image motion vector and photogenic charges transfer, the forward active imaging method utilizes a drift adjusting mechanism rotate the optical focal plane (integrated image sensors) to ensure the same motion direction of both movements. Under the same motion direction, the transfer speed of photo-induced charges in each stage is controlled by the line-transfer signal of photoreceptions to synchronously move with the optical image due to the camera moving. Here, a TDI stage is a row of photo-sensitive elements (See Fig. 1). The line-transfer period of photo-induced charges, matching with the moving time required for the optical line-to-line image, can be expressed as

4 $$T=a\times \frac{H}{f\times {v}_{P}},$$

where *f* is the focal length of the remote sensing camera, *a* is the pixel size of the photoreception, *H* is the distance between the observed objective and the camera, and *v*_*p*_ is the image velocity of optical images. In theory, the drift adjustment mechanism can completely implement the motion direction matching between photo-induced charges and the optical image. However, current drift adjustment mechanisms are physically limited due to a lot of errors, such as control errors, mechanical errors, assembly errors, etc. The optical image direction controlled by the drift adjustment mechanisms couldn’t completely match with the transfer direction of photo-induced charges. On the other hand, the transfer speed of photo-induced charges is determined by *H*, *v*_*P*_, and *f*. The velocity vector (*v*_*p*_, *β*) is calculated using an imaging equation in conjunction with coordinate transformation approaches^12,36^, (*v*_*p*_, *β*) needs all kinds of auxiliary parameters, involving camera positions and attitude parameters from attitude sensors (e.g. gyroscope and GPS). However, these auxiliary parameters for the calculating (*v*_*p*_, *β*) include a lot of errors due to the measurement errors of sensors. In particular, the measurement frequency of current attitude sensors is far lower than the photogenic charges line-transfer frequency, which means mismatching between optical image movements and photogenic charge transferring exists within the period of the integration time. Therefore, motion aberrations disturb dynamic imaging and motion blur exist in acquired images. The dynamic imaging process with an integration time *T* is modeled as^37^:

5 $$g(x,y)={K}_{sensor}(u(x,y)+\eta (x,y)),(x,y)\in {\rm{{\rm B}}}$$

where (*x*, *y*) is the sampling grid of the scene *B*, *K*_*sensor*_ is an amplification factor of image sensors, $u(x,y)$ is a image term, and $\eta (x,y)$ is a noise term. The $u(x,y)$ and $\eta (x,y)$ are independent random variables. The $u(x,y)$ and $\eta (x,y)$ follow respectively the Poisson and Gaussian distributions as:

6 $$u(x,y) \sim P(\lambda {\int }_{0}^{T}f(x-{s}_{x}(t),y-{s}_{y}(t))dt)$$

7 $$\eta (x,y) \sim N(0,{\delta }^{2})$$

8 $${s}_{x}(t)={\int }_{0}^{T}{v}_{px}dt,{s}_{y}(t)={\int }_{0}^{T}{v}_{px}dt$$

where *λ* is the quantum efficiency of the image sensor, the function *f* (·) is the original image. (*s*_*x*_(·), *s*_*y*_(·)) is the motion trajectory of the apparent motion between the scene and image sensors during the integration time. The motion blur is modeled by a linear and shift-invariant operator since (*s*_*x*_(·), *s*_*y*_(·)) is the same for the whole image. The following equation can be expressed as:

9 $$\lambda {\int }_{0}^{T}f(x-{s}_{x}(t),y-{s}_{y}(t))dt=\lambda {\int }_{0}^{T}(f\otimes {\delta }_{(t)})dt=\lambda (f\otimes {\int }_{0}^{T}{\delta }_{(t)}dt)=\lambda (f\otimes h)$$

where $\delta (\,\cdot \,)$ is the Dirac delta function at $({s}_{x}(t),{s}_{y}(t))$, *h*(x, y) is the motion PSF (MPSF), which can be expressed as

10 $$h(x,y)={\int }_{0}^{T}{\delta }_{(t)}dt$$

with

11 $${\int }_{{\rm{\Omega }}}h(x,y)=T,h(x,y) > 0$$

where Ω = R^2^ the two dimensions real coordinate space. Here, we use optical flow neural learning networks to invert motion point spread function to enable computational reconstruction of captured images in conjunction with single digital filters.

### Inversion imaging

**Figure 2:**
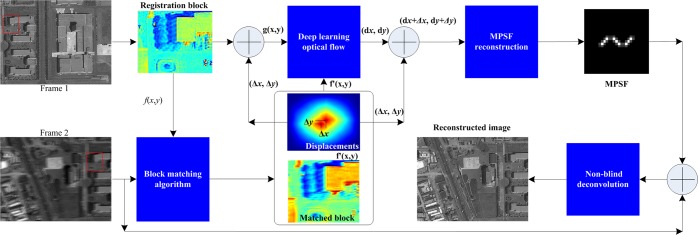
Dynamic inversion imaging using deep learning optical flow. First, the sub-pixel level motion is inverted by deep learning neural networks with motion coarse evaluation of block-matching algorithms. Second, the sub-pixel motion is used to construct the motion PSF with probability density function^49–51^. Third, the motion PSF enables computational reconstruction of captured images with a single digital filter, such as the Wiener filter^52,53^.

The dynamic inversion imaging adopts deep learning optical flows to reconstruct the motion point spread function, enabling removing sub-pixel level motion aberrations. Figure 2 shows the dynamic inversion imaging principle. First, the motion spread function is reconstructed by neural learning networks of optical flow. Two adjacent frame images are used as the input of the motion information extraction. In these two frames, the overlap area is from the same ground objects. Firstly, an image block with the size is *N* × *N* is selected in an overlapped area from the Frame 1, where the selected block area, denoted by *f*(x, y), is called a registration reference template. Then, block registration algorithms^38–40^ are able to apply to the reference template (*f*(x, y)) and Frame 2 to determine the matched block in Frame 2. Here, the matched block in Frame 2 is denoted by *f* ′(x, y). The image motion (denoted by (Δx, Δy)) between the *f*(x, y) and *f* ′(*x*, *y*) can be calculated. The calculated (Δx, Δy) is the pixel-level shifts of the whole block area. Therefore, the block matching method is a coarse motion evaluation and the (Δx, Δy) reflect the integer pixel motion evaluation. Here, we use deep learning optical flow to extract the sub-pixel-level shift.

To extract sub-pixel level movements, we utilize the coarse evaluation of motion (Δx, Δy) calculated by the block registration method. The reference block *f*(*x*, *y*) is firstly shifted by (Δ*x*, Δ*y*) along *x-* and *y*-direction, respectively. A new reference block, denoted by *g*(*x*, *y*), is obtained. The original and new reference block have a relationship of *g*(*x*, *y*) = *f*(*x* + Δ*x*, *y* + Δ*y*). Let the displacements between *g*(*x*, *y*) and *f* ′(*x*, *y*) be (*dx*, *dy*), where the (*dx*, *dy*) is the sub-pixel movement. Thus, the final motion is (Δ*x* + *dx*, Δ*y* + *dy*). The sub-pixel movement (dx, dy) is adjusted on a pixel-by-pixel basis. In this paper, we use learning optical flow to evaluate the (*dx*, *dy*). The new reference block *g*(*x*, *y*) and the matched block *f* ′(*x*, *y*) satisfy the following relationship:

12 $$f^{\prime} (x,y)=g(x+dx,y+dy).$$

Based on the optical flow theory, the *f* ′(*x*, *y*) is expanded into a Taylor series as:

13 $$f^{\prime} (x,y)=g(x,y)+{g^{\prime} }_{x}dx+{g^{\prime} }_{y}dy+R,$$

with

14 $${g^{\prime} }_{x}=\frac{\partial g(x,y)}{\partial x}=g(x+1,y)-g(x,y),$$

15 $${g^{\prime} }_{y}=\frac{\partial g(x,y)}{\partial y}=g(x,y+1)-g(x,y),$$

where *R* is the higher order terms. The optimal movements (*dx*, *dy*) can be evaluated to minimize the cost function as:

16 $$\mathop{{\rm{\min }}}\limits_{dx,dy}{\Vert \sum _{(x,y)}f^{\prime} (x,y)-{\rm{g}}(x,y)-dx\frac{\partial g(x,y)}{\partial x}-dy\frac{\partial g(x,y)}{\partial y}\Vert }^{2}.$$

The deep neural network (DNN) with the back-propagating algorithm can be established to calculate the optimal shift (*dx*, *dy*). In the DNN, the input stimulus is *g*, *g*′_*x*_, *g*′_*y*_; the weights are (*dx*, *dy*); and the output response is *f*′. We use supervised learning^41,42^ to invert sub-pixel-level motion information (*dx*, *dy*) that is considered as the synaptic weights of the deep neural network with multiplayer perceptron. We use weights *w* to express the motion information, i.e. *w* = (*dx*, *dy*). We use *P* with *K* elements to express the input stimulus of the DNN as

17 $$P={\{{P}_{1},{P}_{2},\ldots ,{P}_{k},\ldots \}}_{k=1}^{k=K}={\{g,{g^{\prime} }_{x},{g^{\prime} }_{y}\}}_{K=3}.$$

The desired-response is expressed as *d* = *f*′. Let the training learning task has *J* training examples. The training example set can be expressed as

18 $${\{{S}_{j}\}}_{j=1}^{J}={\{{({P}_{k}(j),d(j))}_{k=1}^{k=K}\}}_{j=1}^{j=J}.$$

**Figure 3:**
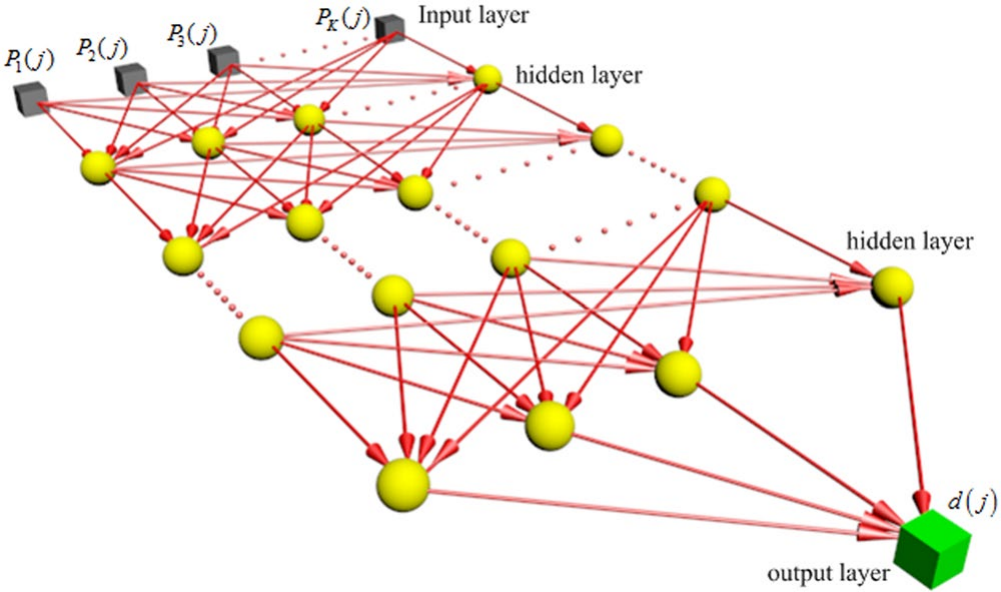
Deep learning network with multiple perceptions for reconstructing optical flow.

Figure 3 shows the deep neural network for reconstructing optical flow. In the DNN, adjustments to synaptic weights, *w*_*jk*_, of the multilayer perception are performed on an example-by-example basis *S*_*j*_. The training examples are arranged in the order *S*_1_, *S*_2_, …, *S*_*J*_, where *J* is the training example number. Each training example has *K* input stimulus. The first example basis *S*_1_ is presented to the DNN, and the weight adjustments are performed with a back-propagation algorithm^43,44^. Then the second example *S*_2_ is presented to the DNN, where the weights are adjusted further. This procedure is continued until the last example *S*_*J*_ is performed.

**Figure 4:**
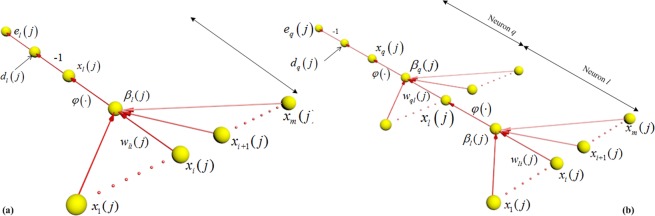
(**a**)The neuron *l* is located in the output layer of the network, (**b**)single node structure of output neuron *q* connected to hidden neuron *l*.

The DNN with multilayer perceptron is achieved by means of two propagating passes, i.e. the forward pass and the backward pass. In the forward pass, the local fields and function (output) signals of the neurons are computed by proceeding forward through the network, layer by layer. It is important to note that the synaptic weights remain unaltered throughout the network, and the function signals of the network are computed on a neuron-by-neuron basis in the forward pass. The backward pass computation starts at the output layer by passing the error signals leftward through the network, layer by layer, and recursively computing the local gradient signals for each neuron. In these two propagating passes, the neurons located on the output and hidden layers have different weight updating and the local gradient forms. Figure 4(a) shows that the neuron *l* is located at the output layer and the hidden layer. When the neuron *l* is a hidden node, the neuron *l* is fed by a set of function (out) signals produced by a hidden layer of neurons to its left in the forward propagating pass. the local filed *β*_*l*_(*j*) of the neuron *l* is calculated with the input of the activation function related to the neuron *l*, which is expressed as:

19 $${\beta }_{l}(j)=\sum _{i=1}^{m}{w}_{li}(j){x}_{i}(j),$$

where *m* is the total number of inputs applied to the neuron *l*. In the output layer, the function signal *x*_*l*_(*j*) produced at the output of neuron *l* by the stimulus input *S*(*j*) is

20 $${x}_{l}(j)={\phi }_{l}({\beta }_{l}(j)).$$

In the backward propagating pass, the corresponding error signal produced at the output of neuron *l* is expressed as:

21 $${e}_{l}(j)={d}_{l}(j)-{x}_{l}(j).$$

The instantaneous error energy of neuron *j* is expressed as:

22 $${{\rm A}}_{l}(j)=\frac{1}{2}{{e}_{l}}^{2}(j).$$

The total instantaneous error energy of the whole network is

23 $${\rm A}(j)=\sum _{l\in C}{A}_{l}(j),$$

where the set *C* consists of all the neurons in the output layer. In the backward propagating pass, the correction weights of the neuron *l* are calculated as:

24 $${\rm{\Delta }}{w}_{li}=-\,{\rho }_{1}\frac{\partial A(j)}{\partial {w}_{li}(j)}={\rho }_{1}{\tau }_{l}(j){x}_{l}(j),$$

where ${\tau }_{l}(j)$ is the local gradient of the neuron *l*, which can be expressed as:

25 $${\tau }_{l}(j)={e}_{l}(j)\frac{\partial {\phi }_{l}({\beta }_{l}(j))}{\partial {\beta }_{l}(j)}.$$

When the neuron *l* is a hidden node, the specified desired response for this neuron doesn’t exist. In the backward propagating pass, the error signal for a hidden neuron is determined recursively and working backward in terms of the error signals of all the neurons to which that hidden neuron is directly connected. Figure 4(b) shows the neuron *l* as a hidden node of the DNN. The instantaneous error energy of neuron *j* is expressed as:

26 $$A(j)=\frac{1}{2}\sum _{q\in C}{{e}_{q}}^{2}(j).$$

In the backward propagating pass, the local gradient ${\tau }_{l}(j)$ for hidden neuron *l* is expressed as:

27 $${\tau }_{l}(j)=\frac{\partial A(j)}{\partial {x}_{l}(j)}\frac{\partial {\phi }_{l}({\beta }_{l}(j))}{\partial {\beta }_{l}(j)}.$$

The partial derivative $\partial A(j)/\partial {x}_{l}(j)$ is calculated backward. The local gradient of the neuron *l* can be expressed as

28 $${\tau }_{l}(j)=\frac{\partial {\phi }_{l}({\beta }_{l}(j))}{\partial {\beta }_{l}(j)}\sum _{q}{\tau }_{q}(j){w}_{ql}(j).$$

The whole DNN for optical flow reconstruction is summarized into the Algorithm 1.

**Algorithm 1:**
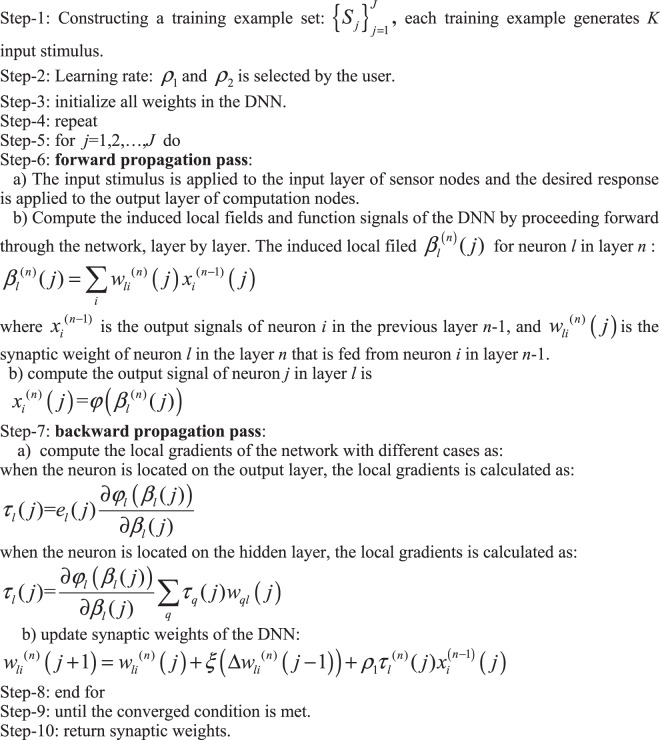
Optical flow reconstruction with deep learning networks.

## Experimental Results

**Figure 5:**
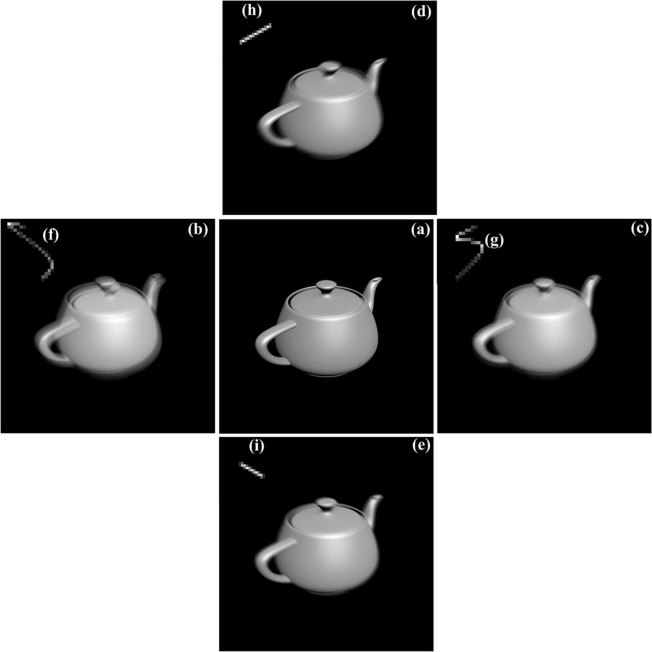
Original image, (**b**–**e**) degraded images, (**f**–**i**) motion point spread function.

**Figure 6:**
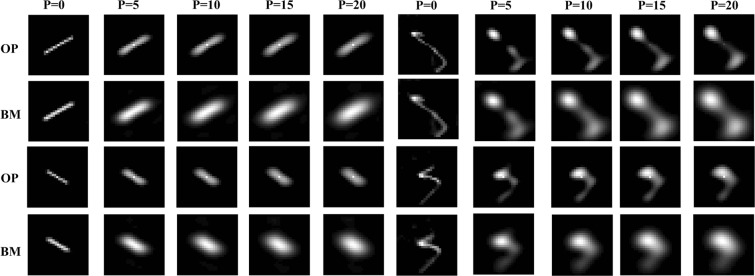
Measured motion PSF at different noise level, OP is the optical flow method and BM is the block matching without optical flow, *P* is the variance of random noise.

To verify the proposed method, we use self-generated images to simulate dynamic inversion imaging. The simulation demonstrates the capability of the proposed method to reconstruct PSF accurately from different motion blurred images. Teapot images are produced in 3ds Max. The self-generated teapot image is blurred with the different motion PSFs (See Fig. 5). The corresponding motion PSFs are directly upper left their blurred images in Fig. 5. Random noise with different noise level (*P*) is added to the blurred images. To verify the proposed method, we use the method with deep learning optical flow and the block matching (without deep learning optical flow) to reconstruct the motion PSF from blurred images. Figure 6 shows the measured motion PSFs using the two methods. The measured results show that the proposed method has more accurate the motion PSF than the case without deep learning optical flow.

**Figure 7:**
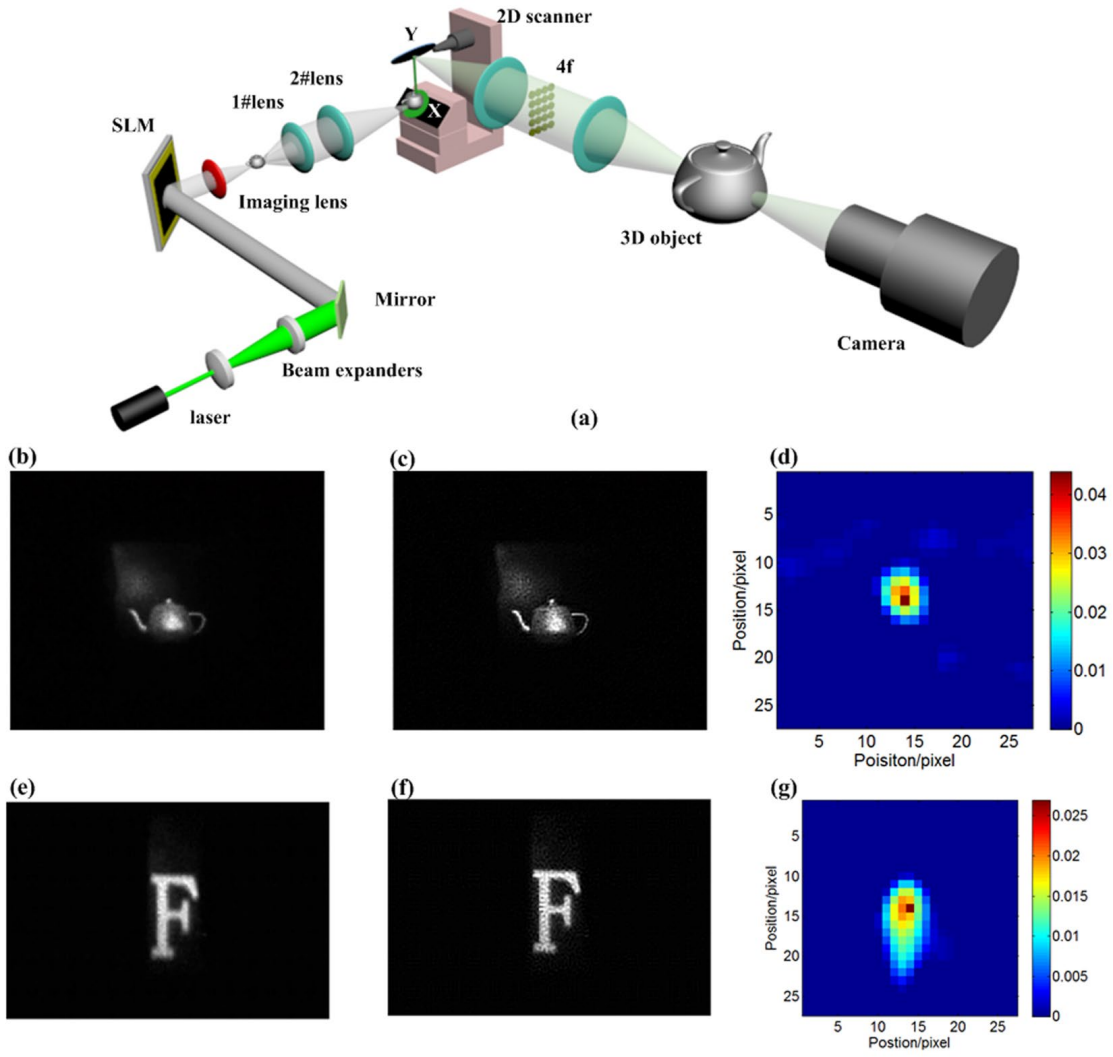
Dynamic imaging experiments, (**a**) setup, (**b**) and (**e**) observed images, (**c**) and (**f**) reconstructed images with the measured motion PSF, (**d**) and (**g**) measured PSF using the proposed method.

We experimentally demonstrate the proposed method to reconstruct the motion PSF of the camera and to remove motion aberrations. A sketch of the dynamic imaging setup is shown in Fig. 7(a). We use a dynamic coarse integration holography^45^ to produce a dynamic 3D objects. A 5 mW point laser with the wavelength of 635 nm is used as the optical source. The laser beam is collimated and expanded by two lenses. The collimated beam is reflected by a mirror and then passes through a spatial light modulator (SLM). After that, the beam is diffracted by the SLM. To implement the dynamic forward imaging, a 2D scanner, and a galvanometric scanner (QS-30) provided by Nutfield Technology, is used to implement the movement of an optical image. The QS-30 has 30 mm and 45 mm aperture mirrors and could handle inertias in the 0.6 to 80 g-cm^2^ range. We use 3ds Max to design a 3D and 2D object model. A layer-based CGH algorithm is used to calculate holograms. The calculated holograms are displayed through the SLM and scanning system. A camera with the focal length of 55 mm, a maximum aperture of *f*/5.6, and pixel size of 4.77um observes the 3D and 2D holographic image. The image sensor of the camera is a CMOS detector; the spectral range is 400-1000 nm. Our method is geared to meet the requirements of the TDI push-broom imaging mode. In our experiments, we use the shutter mode of the CMOS detector to implement the TDI push-broom imaging mode, enabling the same results as a TDI sensor. Figure 7(b,e) show the captured images by the camera. Figure 7(c,f) show the reconstructed image. Figure 7(d,g) show the reconstructed motion PSF. From the inversion imaging results, the proposed method gains the better image quality than the results of the forward imaging.

**Figure 8:**
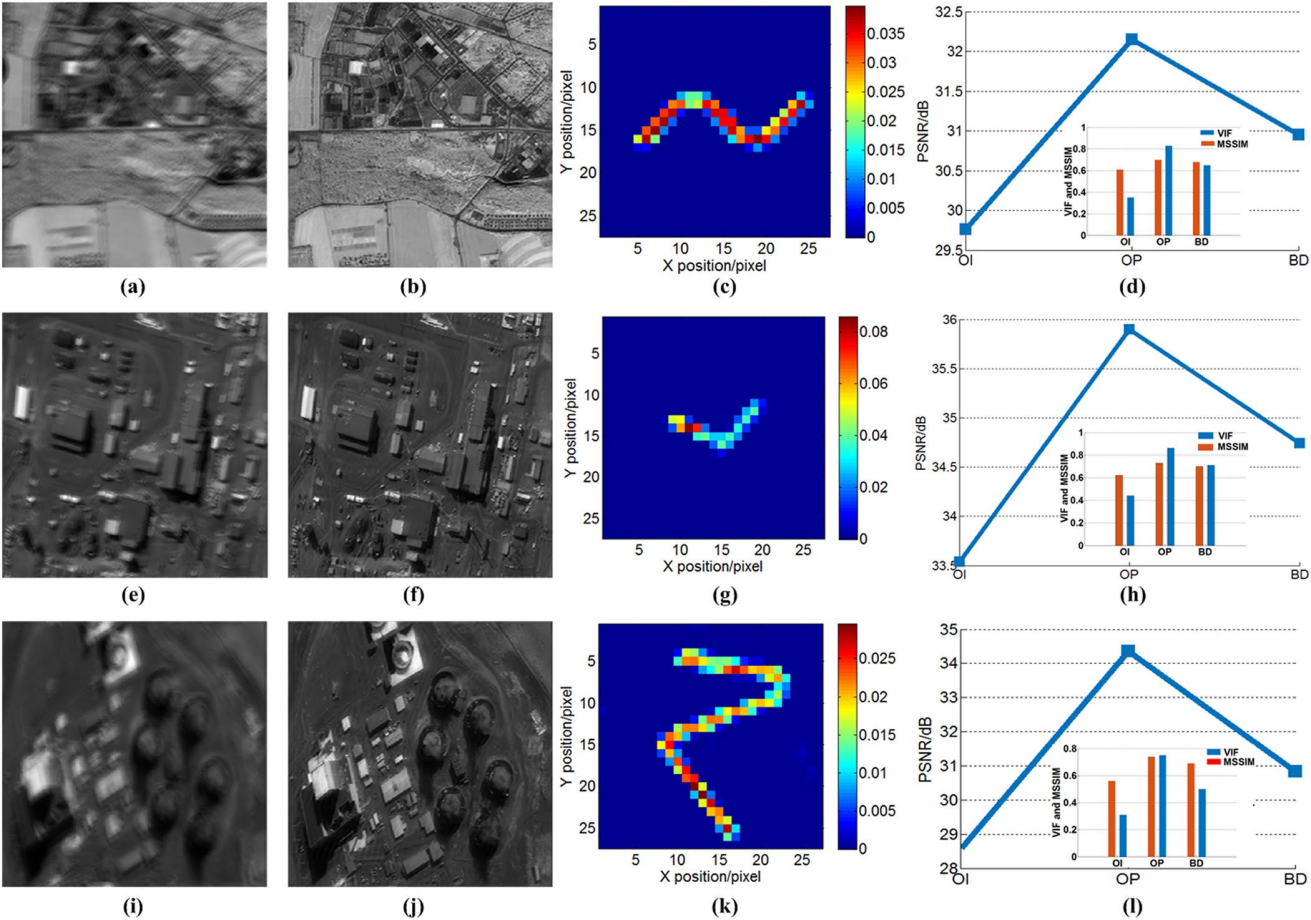
Remote sensing image experiments, blurred images (OI) (**a**,**e** and **i**), reconstructed images with our method (OP) and block matching method (BD) (**b**,**f** and **j**), reconstructed motion PSFs (**c**,**g** and **k**), reconstructed performances (**d**,**h** and **l**).

We also used the remote sensing image to demonstrate the capability of the inversion imaging. The motion PSF is a platform vibration function with a sin function as its basis, which is the motion model widely used in satellite motion. The platform vibration function can be expressed as

29 $$Y(t)=A\,\sin (\frac{2\pi }{{T}_{0}}t+{\alpha }_{0})+N(t),$$

where A is peak value, ${T}_{0}$ is period, ${\alpha }_{0}$ is an original phase, and $N(t)$ is Gauss noise. The motion PSF is constructed based on the proposed method (See Fig. 8(a,g,k)). The measured PSF is used to recover images (See Fig. 8(b,f,j)). After the deconvolution using the motion PSF, the original images can be recovered. The reconstructed images indicate the proposed method has the good results for removing the motion. We also use a mean structural similarity (MSSIM)^46^, peak signal noise ratio (PSNR)^47^, and visual information fidelity (VIF)^48^ to evaluate image performance, which is shown in the Fig. 8(d,h,l). From the measured results, the proposed method gains the better-reconstructed results than the use of the block-matching method without optical flow.

**Figure 9:**
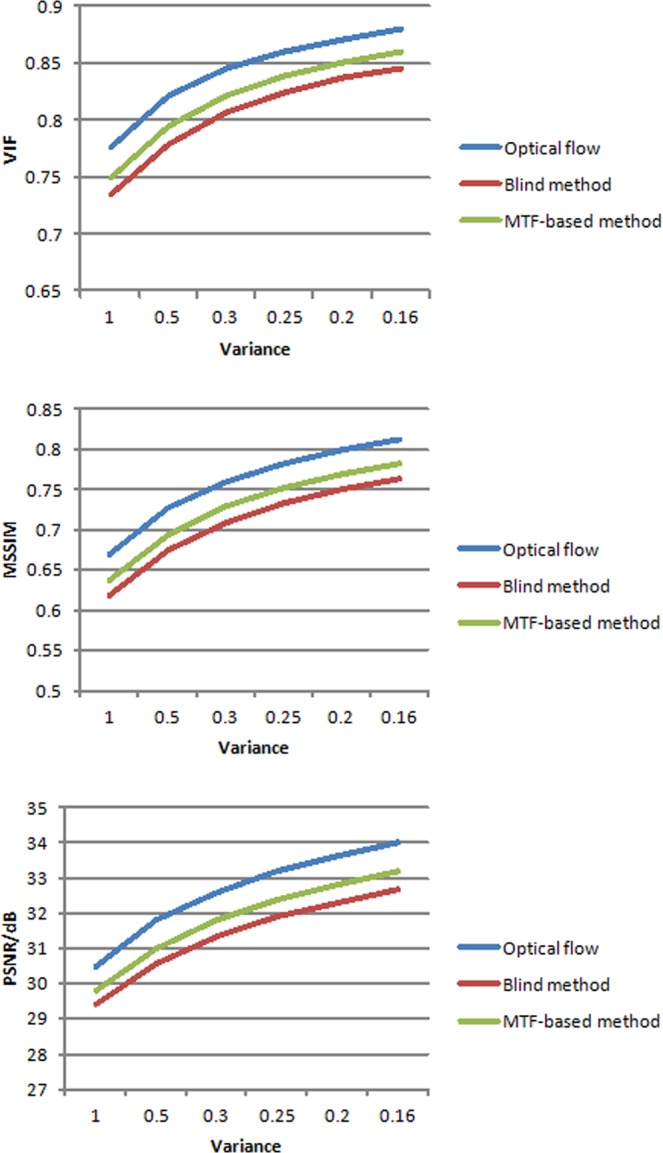
Reconstructed performance results with different approaches.

Finally, we use four images from Satellites to analyze the reconstructed performance. The directly observed images have motion-effects because mismatching of the drift and velocity can result in blurred images. Using the proposed method to remove the motion aberrations, the high-quality image is obtained. We also adopted the blind method and MTF-based method to reconstruct images. We use an MSSIM, VIF and PSNR to evaluate image performance. Figure 9 shows the reconstructed performance of three methods. In Fig. 9, the variance is the different motion deviation from the best motion estimation. In comparing the results, the proposed method has a higher value in PSNR, VIF and MSSIM, which means the proposed has a better-reconstructed result than the other two methods. The proposed method can significantly improve the image quality and remove motion aberrations.

## Conclusion

The motion aberration limits the imaging performance at dynamic conditions due to the mismatching between the image field motion and the photo-induced charge transferring in the forward imaging. This paper reports a novel backward inversion imaging method using deep learning networks of optical flow. First, the optical flow is inverted using deep learning network. Second, the motion point spread function is constructed based on the measured optical flow information. Finally, the motion point spread function computationally reconstructs captured images in conjunction with a single digital filter. This method is experimentally confirmed. This work is a backward and forward imaging link that enables reducing motion aberrations arising from the forward dynamic imaging.
